# Simultaneous Recognition and Relative Pose Estimation of 3D Objects Using 4D Orthonormal Moments

**DOI:** 10.3390/s17092122

**Published:** 2017-09-15

**Authors:** Sergio Dominguez

**Affiliations:** Centre for Automation and Robotics UPM-CSIC, Universidad Politécnica de Madrid, Jose Gutierrez Abascal, 2, 28006 Madrid, Spain; sergio.dominguez@upm.es; Tel.: +34-91-336-3061

**Keywords:** 3D object recognition, relative pose estimation, orthonormal moments

## Abstract

Both three-dimensional (3D) object recognition and pose estimation are open topics in the research community. These tasks are required for a wide range of applications, sometimes separately, sometimes concurrently. Many different algorithms have been presented in the literature to solve these problems separately, and some to solve them jointly. In this paper, an algorithm to solve them simultaneously is introduced. It is based on the definition of a four-dimensional (4D) tensor that gathers and organizes the projections of a 3D object from different points of view. This 4D tensor is then represented by a set of 4D orthonormal moments. Once these moments are arranged in a matrix that can be computed off-line, recognition and pose estimation is reduced to the solution of a linear least squares problem, involving that matrix and the 2D moments of the observed projection of an unknown object. The abilities of this method for 3D object recognition and pose estimation is analytically proved, demonstrating that it does not rely on experimental work to apply a generic technique to these problems. An additional strength of the algorithm is that the required projection is textureless and defined at a very low resolution. This method is computationally simple and shows very good performance in both tasks, allowing its use in applications where real-time constraints have to be fulfilled. Three different kinds of experiments have been conducted in order to perform a thorough validation of the proposed approach: recognition and pose estimation under z axis (yaw) rotations, the same estimation but with the addition of y axis rotations (pitch), and estimation of the pose of objects in real images downloaded from the Internet. In all these cases, results are encouraging, at a similar level to those of state-of-the art algorithms.

## 1. Introduction

Three-dimensional (3D) object recognition and pose estimation are two open problems in the scientific community. There are many algorithms to solve them separately and some to solve them concurrently. In fact, both problems are deeply related, since a 3D object can be observed from different points of view, showing different shapes in each case. Knowing the point of view is equivalent to knowing the relative pose estimation between the object and the observer; once the point of view is known, recognizing the object is much simpler as the observer can explore the set of known objects only under this same point of view. Conversely, knowing the object simplifies pose estimation as well, since the problem is reduced to the exploration of different points of view for this given object.

The complexity of solving both problems increases dramatically when moving from an independent to a concurrent resolution. Therefore, this kind of algorithm is of great value in many applications where object recognition and pose estimation are critical, as is the case of, for instance, object manipulation using a robotic arm.

There have been many proposals to solve each problem separately, since, as stated before, they are critical to many applications. Due to the importance of both topics, reviews are published from time to time regarding them, so there are many sources of information for learning about the state of the art in these two areas.

Recent reviews of available techniques dealing with object recognition can be found in [1] or [2]. A more comprehensive review can be found in [3], or, for a more theoretical point of view, in [4]. Finally, in [5] there is an extensive review of approaches and algorithms.

With respect to pose estimation, the case is much the same, as reviews can be easily found. For instance, in [6] the reader can find an up-to-date review of the most important pose estimation techniques, while in many other sources the reader can find surveys on deeply related techniques as is the case of SLAM [7]. In terms of concurrent solutions of both problems, there have been some remarkable contributions. In [8] the authors introduce an algorithm which uses tensors to generate systems of linear equations that are solved to estimate the affine transformation leading to an object view. In [9], an algorithm using appearance representation is introduced; for a given 3D object, it is coded by extracting brightness information of different views corresponding to different poses. Afterwards, authors perform a dimensionality reduction by applying a PCA to the database. An algorithm for object recognition and pose estimation based on keypoints (SIFT) that builds object models based on pairing keypoints in different views, camera calibration and structure from motion is introduced in [10]. A very similar approach is presented in [11], but in this case no camera calibration is provided. In [12], authors introduce the Viewpoint Feature Histogram, a descriptor based on point clouds that simultaneously encodes geometry and pose, which requires depth information collected with a stereo vision system in order to work. In [13] authors present a probabilistic framework for object recognition, combined with a pose estimation by means of Hu moments applied to the segmented image of tip-shaped objects. In [14] authors combine features describing depth, texture and shape for object recognition, and solve the pose estimation problem by successive steps, using 3D features to find a coarse solution and ICP for fine estimation. In [15], the authors combine 3D CAD descriptions and two-dimensional (2D) texture information to generate a model suitable for the recognition and pose estimation stage, using an approach based on generating multiple virtual snapshots from many different points of view. The authors of [16] introduce an approach based on point cloud alignment, using an RGB-D camera to get a point cloud that is matched against point clouds describing the known 3D objects stored in a database; pose estimation is computed from the alignment process. In [17], authors present an algorithm to estimate the pose of a reduced set of known colored objects; for that purpose, once the colored object is detected in the image and segmented, it is identified by its color and then its 3D model is used to perform a point-of-view sampling process. Finally, in [18], the authors present an algorithm based on template matching and candidate clustering for object recognition and point cloud processing techniques for pose estimation.

In this paper, a new approach to simultaneous object recognition and pose estimation based on the computation of a set of orthonormal moments is introduced. With respect to other methods that use feature spaces or subspaces to solve the task, like for instance [9], this new approach has two main advantages. Firstly, this is an analytic method, with an ability to recognize 3D objects and to estimate their poses as demonstrated in Section 3. For other methods, this ability is a result of experimental work, and therefore is not analytically proved. Second, orthonormal moments are not features in the traditional way, as they capture the object shape itself, not some properties measured on them. In other words, a 3D object can be straightforwardly reconstructed from a complete or truncated series of orthonormal moments, but not from a set of brightness or texture features. Therefore, this algorithm need not be trained each time the database is modified; new objects are simply added by including the orthonormal moment information, formatted in the way that will be explained in Section 3. During an off-line stage a four-dimensional (4D) tensor comprising the information on the 3D object’s shape observed under multiple points of view is built. Additional advantages are that they may be extracted from very low definition images, with a textureless description of detected objects, and that recognition and pose estimation are achieved by means of a matrix product, whose results are straightforwardly interpretable. All these features make it a good candidate for real-time implementations.

The remainder of this paper is organized as follows: related work is reviewed in Section 2. In Section 3 the proposed algorithm is thoroughly explained, first for a simplified case that allows an easier introduction to the underlying concepts where pose estimation is limited to a rotation angle, and after that for the complete case. Section 4 describes how the problem of angular description and balancing has been solved in the proposed approach. In Section 5 experimental results are presented and discussed. Finally, conclusions extracted out of this work are presented in Section 6.

## 2. Related Work

Moments have been used for object recognition and pose estimation, and sometimes for performing both tasks at the same time. Since object recognition is probably the most classical application of moments, the reader is advised to read a comprehensive review on the topic in [4]. Their application for pose estimation is not that extensive, and therefore it is worth mentioning the most significant efforts in this direction. In [19], authors introduce an algorithm to estimate the pose of a satellite by means of computing Zernike moments and comparison with different views of the spacecraft described in the same way. In [20], authors present two alternative methods to estimate the full pose of planar objects, combining the definition of the interaction matrix, central moments, and invariant definition. In [21] authors calculate the moments of a 3D object with known geometry as it moves freely in 3D space, and compile them in a table whose entries are the rotation angles; observed moments are matched against tabulated descriptions. In [22], the author propose invariants to translation, rotation and scaling departing from shifted geometric moments. In [23] the authors propose a method for IBVS based on a set of moments and the definition of an interaction matrix for that set of moments. Finally, there have been some other methods for object recognition based on the characterization of a 3D object by means of the definition of its projections from multiple points of view. Examples of these works are presented by, for example, Ansary et al. [24], who present the selection of multiple views as an adaptive clustering problem; Daras and Axenopoulos [25], who introduce a 3D shape retrieval framework based on the description of an object by means of multiple views; Rusu et al. [12], who introduce an algorithm to recognize an object and estimate its pose using point clouds captured from different points of view; Rammath et al. [26], who describe and recognize cars based on matching 2D and 3D curves that can be observed in different views; Sarkar et al. [15], who present an object recognition algorithm based on feature extraction from a set of computer generated object views; and finally, Liu et al. [27], who introduce a combination of 3D and 2D features obtained from different captures of an object using RGB-D cameras.

## 3. Simultaneous Recognition and Pose Estimation

In this section the derivation of the algorithm for simultaneous 3D object recognition and relative pose estimation is presented. In the first subsection, and for the purpose of explaining the basic ideas on a simpler case, a reduced version is presented, defining a 3D problem in terms of cylindrical coordinates. Afterwards, in the second subsection, the algorithm is generalized to the 4D case, allowing for the determination of an arbitrary relative pose of the observer with respect to the unknown 3D object.

### 3.1. The Problem of a Radial Section in Cylindrical Coordinates

#### 3.1.1. Derivation of the Algorithm

Let the analytic model of a 3D object be expressed as I(ρ,θ,z):Ω↦{0,1}, where Ω stands for the unit cylinder defined as:

(1) Ω={(ρ,θ,z)/ρ∈[0,1],θ∈[0,2π],z∈[0,1]}

with ρ being its radius, θ the angle around its axis, and z its height.

Normalization is required if the original 3D object does not fit and therefore cannot be defined into Ω.

Let the scalar product between a pair of functions in this region be defined as:

(2) $$<x\left(\rho ,\theta ,z\right),y\left(\rho ,\theta ,z\right)>=\int_{\Omega }x\left(\rho ,\theta ,z\right)\bar{y(\rho ,\theta ,z)}\;dv$$

where $\bar{x}$ stands for the complex conjugate of *x* and dv for the differential of volume within Ω.

Given this expression for the scalar product, the pair of functions $B_{nmp}\left(\rho ,\theta ,z\right)$ and $B_{qrs}\left(\rho ,\theta ,z\right)$ are said to be orthonormal in Ω if and only if:

(3) $$<B_{nmp}\left(\rho ,\theta ,z\right),B_{qrs}\left(\rho ,\theta ,z\right)>=\delta_{mq}\delta_{nr}\delta_{ps}$$

where

(4) $$\delta_{ab}=\left\{\begin{array}{cc} 1 & a=b\\ 0 & a\neq b \end{array}\right.$$

In the same region, let the generic basis function for the moment definition be expressed as:

(5) $$B_{nmp}=\Pi_{m}\left(\rho \right)\;Z_{n}\left(z\right)e^{ip\theta }$$

providing that given two different functions of the basis $B_{nmp}\left(\rho ,\theta ,z\right)$ and $B_{qrs}\left(\rho ,\theta ,z\right)$ they are orthonormal, i.e., they behave as expressed in Equation (4).

Given these definitions, the 3D moment of order {m,n,p} of the 3D solid I(ρ,θ,z) with respect to the base function $B_{nmp}\left(\rho ,\theta ,z\right)$ is defined as:

(6) $$V_{nmp}=\frac{1}{\Lambda }\int_{\Omega }I\left(\rho ,\theta ,z\right)\bar{B_{nmp}\left(\rho ,\theta ,z\right)}\;dv=\frac{1}{\Lambda }\int_{\Omega }I\left(\rho ,\theta ,z\right)\Pi_{m}\left(\rho \right)\;Z_{n}\left(z\right)e^{-ip\theta }\;dv$$

where Λ is a normalizing factor that ensures orthonormality.

As the basis functions are orthonormal, the original function defining the 3D solid can be reconstructed from the set of moments by applying the following expression:

(7) $$I\left(\rho ,\theta ,z\right)\approx \sum_{\forall mnp}V_{mnp}B_{mnp}\left(\rho ,\theta ,z\right)=\sum_{\forall mnp}V_{mnp}\Pi_{m}\left(\rho \right)Z_{n}\left(z\right)e^{ip\theta }$$

Given the definition of the 3D function characterizing the volume and the approximation stated in Equation (7), a radial section of the volume can be expressed and approximated in the following way:

(8) $${\left.I(\rho ,\theta ,z)\right|}_{\theta =\theta_{0}}=I\left(\rho ,\theta_{0},z\right)\approx \sum_{\forall mnp}V_{mnp}B_{mnp}\left(\rho ,\theta_{0},z\right)=\sum_{\forall mnp}V_{mnp}\Pi_{m}\left(\rho \right)Z_{n}\left(z\right)e^{ip\theta_{0}}$$

This section is a 2D object defined in the region Ψ={(ρ,z)/ρ∈[0,1],z∈[0,1]}, so on reducing the 3D basis functions to the 2D case by removing the term depending on θ in Equation (5), the 2D moment $S_{kl}$ of this radial section can be defined as:

(9) $$S_{kl}=\int_{\Psi }I\left(\rho ,\theta_{0},z\right)\Pi_{k}\left(\rho \right)Z_{l}\left(z\right)\;dS$$

where dS stands for the differential of surface in Ψ.

Now, replacing in Equation (9) the radial section by its approximation and particularizing for $\theta =\theta_{0}$ in the Equation (8), it can be stated that:

(10) $$S_{kl}\approx \int_{\Psi }\left[\sum_{\forall mnp}V_{mnp}\Pi_{m}\left(\rho \right)Z_{n}\left(z\right)e^{ip\theta_{0}}\right]\Pi_{k}\left(\rho \right)Z_{l}\left(z\right)\;dS=\sum_{\forall mnp}V_{mnp}e^{ip\theta_{0}}\int_{\Psi }\Pi_{m}\left(\rho \right)Z_{n}\left(z\right)\Pi_{k}\left(\rho \right)Z_{l}\left(z\right)\;dS$$

and, taking into account the orthonormality of the basis functions, most of the pairs under the integral sign in Equation (10) cancel out, resulting in:

(11) $$S_{kl}\approx \sum_{\forall mnp}V_{mnp}e^{ip\theta_{0}}\delta_{mk}\delta_{nl}=\sum_{p}V_{klp}e^{ip\theta_{0}}$$

Extending this approximation to a set of moments, with m∈{0…M}, n∈{0…N} and p∈{0…P}, and then reordering 2D moments in a vector, it results in a column vector S with dimensions (M+1)(N+1)×1:

(12) $$S_{(M+1)(N+1)\times 1}={\left[\begin{array}{cccccccc} S_{00} & \ldots & S_{0N} & S_{10} & \ldots & S_{M0} & \ldots & S_{MN} \end{array}\right]}^{T}$$

and 3D moments in the following matrix:

(13) $$V_{\left(M+1)(N+1)\times (P+1\right)}=\left[\begin{array}{cccc} V_{000} & V_{001} & \ldots & V_{00P}\\ \vdots & \vdots & \ddots & \vdots \\ V_{0N0} & V_{0N1} & \ldots & V_{0NP}\\ V_{100} & V_{101} & \ldots & V_{10P}\\ \vdots & \vdots & \ddots & \vdots \\ V_{M00} & V_{M01} & \ldots & V_{M0P}\\ \vdots & \vdots & \ddots & \vdots \\ V_{MN0} & V_{MN1} & \ldots & V_{MNP} \end{array}\right]$$

and finally the angular terms particularized for $\theta =\theta_{0}$:

(14) $$\Gamma_{(P+1)\times 1}=\left[\begin{array}{c} e^{i0\theta_{0}}\\ e^{i1\theta_{0}}\\ \vdots \\ e^{iP\theta_{0}} \end{array}\right]$$

In this way, computing an approximate set of 2D moments of a radial section of a 3D object that has been approximated by the given set of orthonormal 3D moments can be expressed in matrix form as:

(15) S≈V·Γ

Posing the inverse problem, i.e., finding the angle of a radial section of the volume knowing the 3D set of moments and the computed 2D moments of the section (assuming (M+1)(N+1)≥(P+1)) it is reduced to solve a least squares problem expressed by:

(16) $$\Gamma \approx {\left[V^{T}\cdot V\right]}^{-1}V^{T}\cdot S$$

Recalling the structure of vector Γ from Equation (14), the solution to Equation (16) is such that:

(17) $$\log\Gamma =\left[\begin{array}{c} \log(e^{i0\theta_{0}})\\ \log(e^{i1\theta_{0}})\\ \vdots \\ \log(e^{iP\theta_{0}}) \end{array}\right]=i\theta_{0}\left[\begin{array}{c} 0\\ 1\\ \vdots \\ P \end{array}\right]=i\theta_{0}{\left[i-1\right]}_{P+1}$$

where ${\left[i-1\right]}_{P+1}$ stands for a vector with P+1 elements whose *i*-th element takes the value i−1.

#### 3.1.2. Application to Object Recognition and Pose Estimation

Let us consider a problem where a 3D object among a set of *K* elements must be recognized departing from a 2D moment representation of one of its radial sections, that will be called $S_{obs}$. In this scenario, not only is the 3D element unknown, but also the angle $\theta_{0}$ that defines the observed section. Let $V_{k}$ be the 3D moment representation matrix of the *k*-th element of the 3D object set, that has been computed off line. Then, applying Equation (16) to every $V_{k}$ using $S_{obs}$ a corresponding $\Gamma^{k}$ results in:

(18) $$\Gamma_{k}={\left[V_{k}^{T}\cdot V_{k}\right]}^{-1}V_{k}^{T}\cdot S_{obs}$$

A new vector is derived from each $\Gamma_{k}$ in the following way:

(19) $$\Delta_{k}=\frac{\log\Gamma_{k}}{\log\gamma_{2}^{k}}$$

where $\gamma_{2}^{k}$ stands for the second element of the vector $\Gamma_{k}$.

Then, the following distance for every $\Delta_{k}$ is computed:

(20) $$D\left(k\right)=d\left(\Delta_{k},{\left[i-1\right]}_{P+1}\right)$$

where d(u,v) stands for the Euclidean distance between vectors *u* and *v*.

Therefore, the best candidate for the 3D object that has generated the observed section represented by $S_{obs}$ is $k^{*}$ if:

(21) $$D\left(k^{*}\right)=\min_{\forall k}D\left(k\right)$$

and it will be accepted as the actual solution if $D(k^{*})<\tau$, being τ a threshold that is used to prevent false positives.

Once the 3D object has been identified, the angle $\theta_{0}$ that generates the observed section can be computed as well; recalling the structure of Γ from Equation (14), it is easy to see that:

(22) $$\theta_{0}=\left|\log\gamma_{2}^{k^{*}}\right|$$

### 3.2. The Problem of an Arbitrary Projection

#### 3.2.1. Derivation of the Generalized Algorithm

The orthogonal projection of a 3D object in a given direction results in a 2D projection whose definition depends on a set of four coordinates: two angles to define the projection vector and two surface coordinates for the projected 2D object. Therefore, the projection function, which depends on those four parameters, can be defined as P(ϕ,λ,x,z):Ω↦{0,1}, where Ω stands for the hypervolume defined by:

(23) Ω={(ϕ,λ,x,z)/ϕ∈[−π/2,π/2],λ∈[0,2π],x∈[−1,1],z∈[−1,1]}

where the pair (ϕ,λ) stands for the latitude and longitude that defines the projection direction, and the pair (x,z) stands for the Cartesian coordinates where the 2D object that results from the projection by the given direction is defined. It is worth noting that both Cartesian coordinates range from −1 to 1 due to the orthonormality condition in the plane, so the 2D projection has to be normalized to fit into this region.

Note that no roll angle information has been considered in the algorithm. The main reason is that, in most cases, roll is not present as an important part of the relative pose between the object and observer. Nevertheless, in the case of being necessary, it could be incorporated into the algorithm by using a rotation invariant representation for the 2D projection, such as, for instance, Zernike moments on the unit circle.

The function $\chi :R^{4}\mapsto R^{2}$ can be represented by the method of moments as well as other linear, flat or volumetric functions, and the moment basis for this representation can be chosen to be orthonormal, as in the previous case. Let a 4D orthonormal basis in the given coordinates be:

(24) $$B_{mnpq}\left(\phi ,\lambda ,x,z\right)=P_{m}\left(x\right)P_{q}\left(z\right)e^{in\phi }e^{ip\lambda }$$

where $P_{m}\left(x\right)$ stands for the *m*-th element of a family or orthonormal functions in x∈[−1,1], such as, for instance, in Legendre or Tschebychev polynomials; the Fourier basis has been chosen for the transformation of the angular magnitudes (ϕ,λ).

Once again, to generate the moment of order mnpq of the function χ(.), its transformation on the 4D elements of the corresponding moment basis is done by means of the scalar product:

(25) $$H_{mnpq}=\langle \mathrm{Proj}\left(\phi ,\lambda ,x,z\right),B_{mnpq}\left(\phi ,\lambda ,x,z\right)\rangle =\frac{1}{\Phi }\int_{\Omega }\chi \left(\phi ,\lambda ,x,z\right)\bar{B_{mnpq}\left(\phi ,\lambda ,x,z\right)}dhV$$

where Φ stands for a normalization factor, $H_{mnpq}$ stands for the 4D moment, and dhV stands for the differential of the hypervolume in Ω.

As in Equation (7), since functions in the base are orthonormal, it is possible to build a reconstruction of the 4D function using the computed moments:

(26) $$\chi \left(\phi ,\lambda ,x,z\right)\approx \sum_{\forall \{mnpq\}}{\left[HB\right]}_{mnpq}\left(\phi ,\lambda ,x,z\right)$$

where ${\left[HB\right]}_{mnpq}\left(\phi ,\lambda ,x,z\right)=H_{mnpq}B_{mnpq}\left(\phi ,\lambda ,x,z\right)$.

In this way, a projection of the 3D object through a given direction $\left(\phi_{0},\lambda_{0}\right)$ can be approximated by:

(27) $${\left.\chi (\phi ,\lambda ,x,z)\right|}_{\left\{\phi_{0},\lambda_{0}\right\}}\approx \sum_{\forall \{mnpq\}}{\left[HB\right]}_{mnpq}\left(\phi_{0},\lambda_{0},x,z\right)=\sum_{\forall \{mnpq\}}H_{mnpq}P_{m}\left(x\right)P_{q}\left(z\right)e^{in\phi_{0}}e^{ip\lambda_{0}}$$

The 2D moments of this projection will be given, as in Equation (9), by:

(28) $$S_{kl}=\int_{\Psi }\chi \left(\phi_{0},\lambda_{0},x,z\right)P_{k}\left(x\right)P_{l}\left(z\right)\;dS$$

being Ψ={(x,z)/x∈[−1,1],z∈[−1,1]}.

Replacing the analytic expression of the projection function by its 4D moment- based approximation:

(29) $$S_{kl}\approx \int_{\Psi }\left[\sum_{\forall \{mnpq\}}{\left[HB\right]}_{mnpq}^{\phi_{0}\lambda_{0}}\right]P_{k}\left(x\right)P_{l}\left(z\right)\;dS=\sum_{\forall \{mnpq\}}\int_{\Psi }{\left[HB\right]}_{mnpq}^{\phi_{0}\lambda_{0}}P_{k}\left(x\right)P_{l}\left(z\right)\;dS$$

where ${\left[HB\right]}_{mnpq}^{\phi_{0}\lambda_{0}}=H_{mnpq}B_{mnpq}\left(\phi_{0},\lambda_{0},x,z\right)$. Then, following the same reasoning as in Equation (11):

(30) $$S_{kl}\approx \sum_{\forall \{mnpq\}}H_{mnpq}e^{in\phi_{0}}e^{ip\lambda_{0}}\delta_{mk}\delta_{lq}=\sum_{\forall \{np\}}H_{mnpq}e^{in\phi_{0}}e^{ip\lambda_{0}}$$

As in the previous section, extending this approximation to the whole set of 2D moments, with m∈[0…M], n∈[0…N], p∈[0…P] and q∈[0…Q] and reordering 2D moments in a vector and 4D moments in a matrix, the following matrices can be defined:

(31) $$H_{(M+1)(Q+1)\times 1}={\left[\begin{array}{cccccc} H^{00} & H^{01} & \cdots & H^{0Q} & \cdots & H^{MQ} \end{array}\right]}^{T}$$

where

(32) $$H_{1\times (N+1)(P+1)}^{mq}=\left[\begin{array}{ccccccccc} H_{mq00} & H_{mq01} & \cdots & H_{mq0P} & H_{mq10} & \cdots & H_{mq1P} & \cdots & H_{mqNP} \end{array}\right]$$

so H is a (M+1)(Q+1)×(N+1)(P+1) matrix containing the complete set of 4D moments.

In addition:

(33) $$\Gamma_{(N+1)(P+1)\times 1}={\left[\begin{array}{ccccccccc} e^{i0\phi_{0}}e^{i0\lambda_{0}} & e^{i0\phi_{0}}e^{i1\lambda_{0}} & \cdots & e^{i0\phi_{0}}e^{iP\lambda_{0}} & e^{i1\phi_{0}}e^{i0\lambda_{0}} & \cdots & e^{i1\phi_{0}}e^{iP\lambda_{0}} & \cdots & e^{iN\phi_{0}}e^{iP\lambda_{0}} \end{array}\right]}^{T}$$

Then, the vector containing the set of 2D moments S, defined in Equation (12), of a projection of a 3D object through the direction defined by the pair $\left(\phi_{0},\lambda_{0}\right)$ can be expressed as:

(34) S≈H·Γ

Therefore, given a projection of a known 3D object, whose 2D moments are in S, the matrix containing the angular terms particularized for the pair $\left(\phi_{0},\lambda_{0}\right)$ that defines the projection can be computed as:

(35) $$\Gamma \approx {\left[H^{T}\cdot H\right]}^{-1}H^{T}\cdot S$$

whenever (M+1)(Q+1)≥(N+1)(P+1).

It is straightforward that the solution to Equation (35) be transformed as:

(36) $$\log\Gamma =\left[\begin{array}{c} \log\left(e^{i0\phi_{0}}e^{i0\lambda_{0}}\right)\\ \log\left(e^{i0\phi_{0}}e^{i1\lambda_{0}}\right)\\ \vdots \\ \log\left(e^{i0\phi_{0}}e^{iP\lambda_{0}}\right)\\ \log\left(e^{i1\phi_{0}}e^{i0\lambda_{0}}\right)\\ \vdots \\ \log\left(e^{i1\phi_{0}}e^{iP\lambda_{0}}\right)\\ \vdots \\ \log\left(e^{iN\phi_{0}}e^{iP\lambda_{0}}\right) \end{array}\right]=\left[\begin{array}{c} 0\left(i\phi_{0}\right)+0\left(i\lambda_{0}\right)\\ 0\left(i\phi_{0}\right)+1\left(i\lambda_{0}\right)\\ \vdots \\ 0\left(i\phi_{0}\right)+P\left(i\lambda_{0}\right)\\ 1\left(i\phi_{0}\right)+0\left(i\lambda_{0}\right)\\ \vdots \\ 1\left(i\phi_{0}\right)+P\left(i\lambda_{0}\right)\\ \vdots \\ N\left(i\phi_{0}\right)+P\left(i\lambda_{0}\right) \end{array}\right]$$

Therefore, given $\Gamma_{P}$, that is built taking the first *P* elements of Γ:

(37) $$\log\Gamma_{P}=\left[\begin{array}{c} 0\left(i\phi_{0}\right)+0\left(i\lambda_{0}\right)\\ 0\left(i\phi_{0}\right)+1\left(i\lambda_{0}\right)\\ \vdots \\ 0\left(i\phi_{0}\right)+P\left(i\lambda_{0}\right) \end{array}\right]=i\lambda_{0}\left[\begin{array}{c} 0\\ 1\\ \vdots \\ P \end{array}\right]$$

and $\Gamma_{N}$, that is built taking the elements in positions 1+n(P+1), n∈{0,N}, of Γ is:

(38) $$\log\Gamma_{N}=\left[\begin{array}{c} 0\left(i\phi_{0}\right)+0\left(i\lambda_{0}\right)\\ 1\left(i\phi_{0}\right)+0\left(i\lambda_{0}\right)\\ \vdots \\ N\left(i\phi_{0}\right)+0\left(i\lambda_{0}\right) \end{array}\right]=i\phi_{0}\left[\begin{array}{c} 0\\ 1\\ \vdots \\ N \end{array}\right]$$

#### 3.2.2. Application to Object Recognition and Pose Estimation

Now, let us suppose that the problem is extended to the determination, not only of the pair of angles defining the projection direction, but also to recognize the 3D object among a given set of *K* different elements whose projection is observed. In this case, let $S_{obs}$ be the set of 2D moments extracted from the observed projection, and $H_{k}$ the 4D moment matrix corresponding to the *k*-th element of the 3D object set. Applying the expression in Equation (35) to every $H_{k}$, a corresponding $\Gamma^{k}$ will be found:

(39) $$\Gamma^{k}={\left[H_{k}^{T}H_{k}\right]}^{-1}H_{k}^{T}S_{obs}$$

Then, for each $\Gamma^{k}$, the following pair of vectors can be built:

(40) $$\Delta_{P}^{k}=\frac{\log\Gamma_{P}^{k}}{\log\gamma_{2}^{k}},\;\Delta_{N}^{k}=\frac{\log\Gamma_{N}^{k}}{\log\gamma_{P+2}^{k}}$$

where $\gamma_{2}^{k}$ and $\gamma_{P+2}^{k}$ correspond to the second and (P+2)-th elements of vector $\Gamma^{k}$.

In order to find out which is the 3D object that is observed in the given 2D projection, the following expression is computed for each candidate *k*:

(41) $$D\left(k\right)=u\;d\left(\Delta_{N}^{k},{\left[i-1\right]}_{N+1}\right)+v\;d\left(\Delta_{P}^{k},{\left[i-1\right]}_{P+1}\right)$$

where again d(u,v) stands for the Euclidean distance among vectors *u* and *v*, ${\left[i-1\right]}_{X}$ is a vector with *X* elements whose *i*-th element takes the value i−1, and (u,v) are a pair of scalars used to give weights to the estimation for each angle in the pair (ϕ,λ).

Then, the best candidate for being the observed 3D object is $k^{*}$ if:

(42) $$D\left(k^{*}\right)=\min_{\forall k}D\left(k\right)$$

and it will be accepted as the solution if $D(k^{*})<\tau$, where τ is a threshold used to reject false positives.

Once the 3D object has been identified, recovering the pair $\left(\phi_{0},\lambda_{0}\right)$ that defines the projection direction is straightforward; recalling the structure of Γ from Equation (33) it is easy to see that:

(43) $$\phi_{0}=|\log\gamma_{P+2}^{k^{*}}|,\;\lambda_{0}=\left|\log\gamma_{2}^{k^{*}}\right|$$

It is worth highlighting that although obtaining 4D moments is a computationally demanding step, as is the computation of the pseudoinverse for each $H_{k}$, both are computed off-line; therefore, the object identification and the latitude and longitude determination are achieved by the product of a precomputed matrix with a vector, and computing two Euclidean distances, making this algorithm well suited for on-line applications. The more complex on-line computation is due to calculating the observed moments vector that can be obtained in real time as has been demonstrated in the literature, for instance in [28].

## 4. Section vs. Projection: Projected Pseudo-Volume

In Section 3.1 it has been proven that, departing from the orthogonal moment representation of an unknown section of a 3D object in a given set, it is possible not only to recognize what the original object is, but also the angle determining that section. Nevertheless, in any application a common situation is not having the image of a section but of a projection of the whole object that will be assumed to be parallel to a given direction, as is the case of the Radon transform. Yet, in Section 3.2 it is supposed that the projection through a given direction is already defined.

Therefore, in order to make the algorithm work with projections instead of with sections, a transformation of the 3D object must be carried out. This transformation will yield another 3D object gathering all the considered projections through directions at different θ angles for the case of cylindrical coordinates, and will yield a 4D tensor comprising projections from every pair $\left(\phi_{0},\lambda_{0}\right)$ in the case of considering any projection direction.

**Figure 1:**
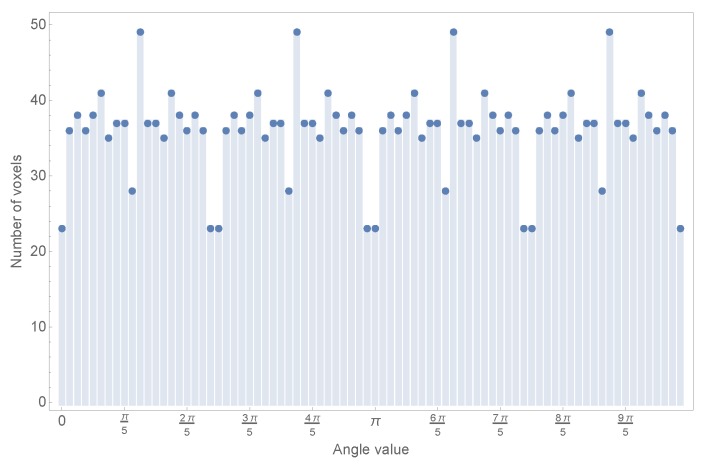
Histogram of angle values in a 60 × 60 grid.

However, before starting with the definition of these transformed objects, it is necessary to study the problem of the polar translation of a Cartesian object in a discrete domain. The main problem is that when this object defined in a Cartesian discrete grid has to be defined through polar coordinates used in the derivation of the algorithm for both cases, the resulting representation is unbalanced in the angular coordinates. To illustrate this point a simple test has been conducted: a simple grid of 60 × 60 pixels has been defined, and the angles of every pixel with a radius under or equal to 60 have been collected in a histogram, where each bin has a width of π/40 rad. The result of this simple test can be seen in Figure 1.

As can be easily observed, the numbers of pixels assigned to each bin are clearly unbalanced. This causes different representation strengths for different angles, affecting the moment transformation as it is based on angular coordinates.

In the recognition stage, the unknown projection image will be represented within a normalized flat grid, with a fixed number of pixels, but, as can be seen from Figure 1, each section for every known object is represented with a varying number of pixels depending on the angle. Obviously, this mismatch will cause the algorithm to perform poorly.

Therefore, as the problem stated herein is defined in angular coordinates, some strategy must be provided in order to balance the pixels contributing to each angle bin, as there is no strength mismatch between unknown projection and object representation.

The solution adopted for this case consists of building a transformed pseudo-volume that packs all these projections into a Cartesian grid, though some axes describe the angles at which the object has been rotated in order to generate a given projection. The construction of this pseudo-volume for cylindrical coordinates is as follows. Let the rotation of the object I(ρ,θ,z) by an angle $\theta_{i}$ around the *z* axis be defined as:

(44) $$I_{\theta_{i}}\left(\rho ,\theta^{\prime },z\right)=I\left(\rho ,\theta +\theta_{i},z\right):\Omega \mapsto \left\{0,1\right\}$$

so the rotated object is defined in the same domain as the original object, Ω, defined in Equation (1), and $\theta_{i}\in \left[0,\theta_{\max}\right]$ , $\theta_{\max}<\pi$. The upper limit for $\theta_{i}$ will be explained later on.

In order to make the next steps clearer, the rotated object is now converted to Cartesian coordinates:

(45) $$I_{\theta_{i}}\left(\rho ,\theta^{\prime },z\right)\to I_{\theta_{i}}\left(x,y,z\right),\left\{\begin{array}{c} x=\rho \cos\theta^{\prime }\\ y=\rho \sin\theta^{\prime }\\ z=z \end{array}\right.$$

where $I_{\theta_{i}}\left(x,y,z\right):\Omega^{*}\mapsto \left\{0,1\right\}$, as

(46) $$\Omega^{*}=\left\{\left(x,y,z\right)/x\in \left[-1,1\right],y\in \left[-1,1\right],z\in \left[0,1\right]\right\}$$

as a result of the change in the coordinate system.

Now, after the object has been rotated, its projection through the *y* axis is generated:

(47) $${\mathrm{PI}}_{\theta_{i}}\left(x,z\right)=\int_{-1}^{1}I_{\theta_{i}}\left(x,y,z\right)\;dy,\;\forall \left\{x\in \left[-1,1\right],z\in \left[0,1\right]\right\}$$

This projected image, ${\mathrm{PI}}_{\theta_{i}}\left(x,z\right)$, is then binarized to bring it back to its original range, i.e., {0,1}:

(48) $${\hat{\mathrm{PI}}}_{\theta_{i}}\left(x,z\right)=\left\{\begin{array}{cc} 0 & {\mathrm{PI}}_{\theta_{i}}\left(x,z\right)=0\\ 1 & {\mathrm{PI}}_{\theta_{i}}\left(x,z\right)\neq 0 \end{array}\right.$$

Extending this process to every $\theta_{i}\in \left[0,\theta_{\max}\right]$, and gathering all the resulting binarized projections, a new 3D object, called the projected pseudo-volume, $\mathrm{PV}\left(x,z,\theta \right):\tilde{\Omega }\mapsto \left\{0,1\right\}$, is generated:

(49) $$\mathrm{PV}\left(x,z,\theta \right)={\hat{\mathrm{PI}}}_{\theta }\left(x,z\right)$$

being:

(50) $$\tilde{\Omega }=\left\{\left(x,z,\theta \right)/x\in \left[-1,1\right],z\in \left[0,1\right],\theta \in \left[0,\theta_{\max}\right]\right\}$$

Once the new 3D object, PV(x,z,θ), is defined, the upper limit for rotation angles, $\theta_{\max}$, can be explained; it is set in order to avoid that any projection, or its reflected image, be represented more than once, as this fact would lead to multiple solutions in a pose estimation problem. Therefore, this upper limit will have a maximum value of $\theta_{\max}<\pi$, as both sides of any object have the same (reflected) projections. In case of symmetric objects this upper limit will have to be reduced in order to avoid a multiplicity of projections within PV(x,z,θ). The limit case corresponds to objects having central symmetry around its *z* axis, for which θ pose estimation cannot be computed as the projections from every rotation angle are the same.

**Figure 2:**
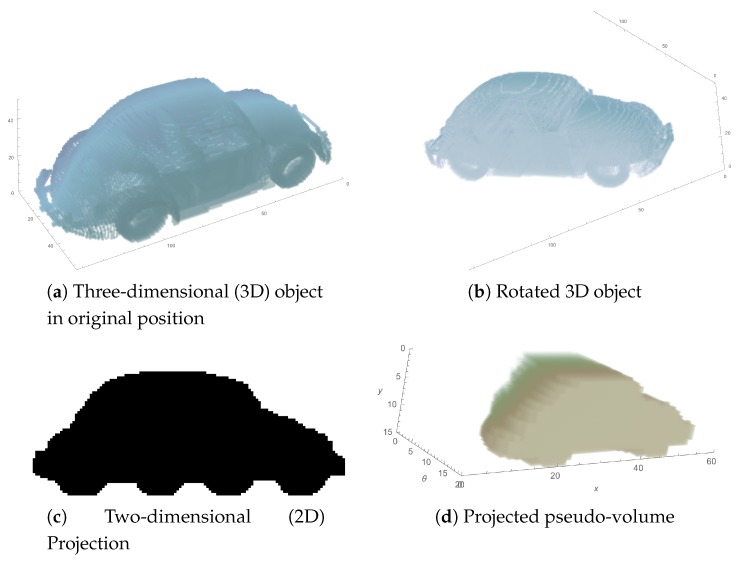
Generation of the projected pseudo-volume: (**a**) Original 3D object, I(ρ,θ,z); (**b**) Object rotated at the $\theta_{i}$ angle around the *z* axis and converted to Cartesian coordinates, $I_{\theta_{i}}\left(x,y,z\right)$; (**c**) Binarized projection through the *y* axis, ${\hat{\mathrm{PI}}}_{\theta_{i}}\left(x,z\right)$; (**d**) Complete PV(x,z,θ) object, after gathering all the projections.

The process of generating this transformed object is resumed in Figure 2. It starts with the 3D object with no rotation (a), i.e., with an initial alignment with *x* and *y* axes, and then it is rotated $\theta_{i}$ around the *z* axis (b). The resulting image has shifted its alignment with the original axes, so once it is projected through the *y* axis, the result will be different for each rotation (c). This projection is included in the new object, as a slice with coordinate value $\theta_{i}$ on the θ axis (d).

In this way, the projected pseudo-volume, that has the same number of pixels assigned to each angle view, collects information about parallel projections in the sense of a Radon transform, instead of simple object sections.

The same idea has been applied to the generation of a 4D object that will be used in the general case as depicted in Section 3.2.

Starting from the initial definition of the 3D object, I(ρ,θ,z) in Ω, it is initially converted to Cartesian coordinates to make the next steps clearer, using an expression similar to Equation (45). As a result, the object is now defined in $\Omega^{*}$, described in Equation (46). Then, the 3D object is rotated an angle $\phi_{i}$ around the *x* axis, so it is now represented as:

(51) $$I_{\phi_{i}}\left(\hat{x},\hat{y},\hat{z}\right),\;\left[\begin{array}{c} \hat{x}\\ \hat{y}\\ \hat{z} \end{array}\right]=\left[\begin{array}{ccc} 1 & 0 & 0\\ 0 & \sin\phi_{i} & \cos\phi_{i}\\ 0 & \cos\phi_{i} & -\sin\phi_{i} \end{array}\right]\left[\begin{array}{c} x\\ y\\ z \end{array}\right]$$

After that, the object is rotated an angle $\lambda_{j}$ around the *z* axis, so the object is redefined as:

(52) $$I_{\phi_{i},\lambda_{j}}\left(\tilde{x},\tilde{y},\tilde{z}\right),\;\left[\begin{array}{c} \tilde{x}\\ \tilde{y}\\ \tilde{z} \end{array}\right]=\left[\begin{array}{ccc} \cos\lambda_{j} & -\sin\lambda_{j} & 0\\ \sin\lambda_{j} & \cos\lambda_{j} & 0\\ 0 & 0 & 1 \end{array}\right]\left[\begin{array}{c} \hat{x}\\ \hat{y}\\ \hat{z} \end{array}\right]$$

From this point on, the algorithm progresses as in the previous case, starting from the projection through the *y* axis, as in Equation (47):

(53) $${\mathrm{PI}}_{\phi_{i},\lambda_{j}}\left(x,z\right)=\int_{-1}^{1}I_{\phi_{i},\lambda_{j}}\left(\tilde{x},\tilde{y},\tilde{z}\right)\;d\tilde{y},\;\forall \left\{\tilde{x}\in \left[-1,1\right],\tilde{z}\in \left[0,1\right]\right\}$$

then there is binarization to bring the image back to its original range, as in Equation (48):

(54) $${\hat{\mathrm{PI}}}_{\phi_{i},\lambda_{j}}\left(x,z\right)=\left\{\begin{array}{cc} 0 & {\mathrm{PI}}_{\phi_{i},\lambda_{j}}\left(x,z\right)=0\\ 1 & {\mathrm{PI}}_{\phi_{i},\lambda_{j}}\left(x,z\right)\neq 0 \end{array}\right.$$

and, finally, extending the rotation process to every $\phi_{i}\in \left[\phi_{\min},\phi_{\max}\right]$ and $\lambda_{j}\in \left[0,\lambda_{\max}\right]$ and gathering all the resulting projections in a new 4D object, the so-called projected pseudo-hypervolume $PH\left(x,z,\phi ,\lambda \right):\tilde{\Omega }\mapsto \left\{0,1\right\}$ is generated:

(55) $$\mathrm{PH}\left(x,z,\phi ,\lambda \right)={\hat{\mathrm{PI}}}_{\phi ,\lambda }\left(x,z\right)$$

where,

(56) $$\tilde{\Omega }=\left\{\left(x,z,\phi ,\lambda \right)/x\in \left[-1,1\right],z\in \left[0,1\right],\phi \in \left[\phi_{\min},\phi_{\max}\right],\lambda_{j}\in \left[0,\lambda_{\max}\right]\right\}$$

## 5. Experimental Results

### 5.1. Recognition and Pose Estimation in Cylindrical Coordinates

For testing this simple case, prior to testing the complete algorithm with the 4D objects described in the previous section, a set of 70 three-dimensional objects out of the the Pascal3D+ Benchmark set [29] has been compiled, belonging to every included class but `Bottle’, since it has central symmetry and therefore is ill-posed for pose estimation. These 70 objects have been transformed from their original CAD format to a voxel-based representation, and are included in a grid of 60×60×15 (width, depth, height) voxels, in such a way that the object’s centroid is placed in the center of the grid, and it is touching some of the grid walls.

Once all these objects have been normalized in this way, a projected pseudo-volume has been defined for all of them, covering 20 equally spaced rotations between 0 and π/2 radians. The angle limit has not been extended to 2π because of the observed four-fold symmetry: symmetric objects produce the same projection for different rotation angles (views), leading the algorithm to the impossibility of choosing the right orientation. In mathematical terms, symmetry leads the presented algorithm to ill-posedness, so the pseudoinverse suffers from numeric instability due to a very bad condition number. In this way, the pseudo-volume generated for each original 3D object is defined in a grid of 60×15×20 (x,z,θ).

For every projected pseudo-volume in the collection, the orthogonal moment representation for each coordinate is by:

- Legendre moments up to the 19th order for the Cartesian coordinates *x* and *z*. The Legendre polynomial of order *n* in *x* is defined as:
  (57) Pn(x)=12n∑k=0nnk2(x+1)n−k(x−1)k
- Fourier base. Note that although this coordinate is defined in a Cartesian grid, it represents an angle in the real world so it can be easily transformed using the Fourier basis that on the other hand allows this algorithm to determine the rotation angle of the observed pose. The Fourier base is up to 19th order for θ as well, i.e.,:
  (58) $$B_{mnp}=P_{m}\left(x\right)P_{n}\left(z\right)e^{ip\theta }$$

In this way, each pseudo-volume has been represented with 8000 descriptors with a total of 18,000 voxels in the pseudo-volume that represent a 3D object initially defined through 54,000 voxels. All these data have been gathered in Table 1.

Once the moments for each object have been generated and stored in the database, a set of unknown side views is generated. For this purpose, each of the 70 objects has been rotated 10 times at random angles in the interval θ∈[0,π/2] and their projections along the *y* axis have been computed. Each projection has been represented through its Legendre moments up to the 19th order in *x* and *z*.

Using this unknown set, two different tests have been carried out. First, a test was run to determine the precision of the algorithm recognizing the object that generated each projection after rotation. Then, a second test has been conducted to evaluate the accuracy, estimating the rotation angle that has been used to generate each projection.

#### 5.1.1. Recognition Tests

Two different rounds of precision tests have been conducted. In the first one, the orders of *x* and *z* moments have been varied from 0 to 19, keeping the order of moments on θ fixed to 19. Mathematically, this means that calling back the general expression in Equation (16), matrix V has been reduced in terms of its number of rows, keeping constant its number of columns, i.e., the least squares problem to be solved has been defined using fewer equations. This fact can be easily seen going back to the definition of matrix V in Equation (13). In this way, for this round the problem has been reformulated in reduced versions through:

(59) $$\Gamma \left(19\right)={\left[V{\left(m,n,19\right)}^{T}\cdot V\left(m,n,19\right)\right]}^{-1}V{\left(m,n,19\right)}^{T}\cdot S\left(m,n\right),\;m,n\in \left[0\ldots 19\right]$$

where V(m,n,19) stands for a matrix V constructed using moments for *x* and *z* up to order *m* and *n* respectively. Also, according to the general expression in Equation (13), S(m,n) stands for a matrix of 2D moments constructed up to orders *m* and *n* as well. Finally, Γ(19) stands for a matrix of Fourier basis elements up to order 19, and is particularized for $\theta =\theta_{0}$, where $\theta_{0}$ is the unknown angle used for generating the projection image under analysis.

In a second round of tests the orders of the moments in *x* and *z* were fixed to 19, and the order in θ was varied from 0 up to 19. Mathematically, this means that the least squares problem in Equation (16) has been defined by always using the same number of equations given by the constant number of rows in matrix V, but with an increasing number of unknowns to be estimated, given by the varying number of columns and the corresponding number of elements in vector Γ(p):

(60) $$\Gamma \left(p\right)={\left[V{\left(19,19,p\right)}^{T}.V\left(19,19,p\right)\right]}^{-1}V{\left(19,19,p\right)}^{T}.S\left(19,19\right),\;p\in \left[0\ldots 19\right]$$

Before starting both rounds of tests, 2D moments for each unknown projection image have been computed, obtaining $S_{obs,u},\;u\in \left[1\ldots 700\right]$. After that, tests have run in the following way:

- First, the order of moments in *x*, *y* and θ is set.
- Matrix $V_{k}$ is reconfigured according to those moment orders to $V{\left(m,n,p\right)}_{k}$.
- Next, $\Gamma {\left(p\right)}_{k,u}$ is computed, according to Equations (59) or (60).
- Vector $S{\left(m,n\right)}_{k,u}$ is computed for each object in the database:
  (61) $$S_{k,u}=V_{k}\Gamma_{k,u}$$
- The |RMS| is computed between the observed and estimated sets of moments:
  (62) $${\left|\mathrm{RMS}\right|}_{k,u}=\left|\sqrt{\frac{1}{\left(m+1)(n+1\right)}\sum_{i=0}^{\left(m+1)(n+1\right)}{\left(S_{k,u}\left(i\right)-S_{obs,u}\left(i\right)\right)}^{2}}\right|$$
- The object $k^{*}$ is obtained, for which the minimum RMS is chosen from the database:
  (63) $${\left|\mathrm{RMS}\right|}_{k^{*},u}=\min_{k,u}{\left(|\mathrm{RMS}\right|}_{k,u})$$
- The *u*-th projection image is recognized as having been generated by object $k^{*}$

According to this test procedure the total number of pairs (k,u) that has been tested is easily computed: 700 unknown images, in two rounds of 20 (m,n,p) combinations. Ten comparisons for each combination results in 700×(2×20)×10= 280,000 combinations.

**Figure 3:**
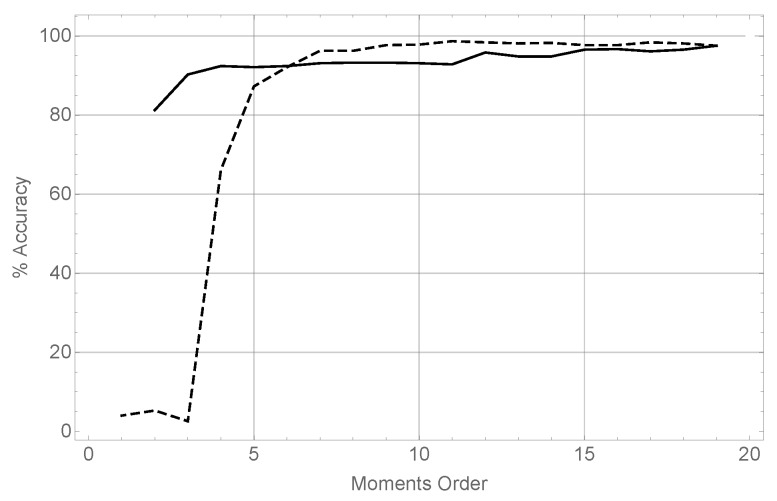
Results of the precision in the recognition tests. The dashed line represents varying moment order in θ while keeping the orders in *x* and *z* constant in 19. The solid line represents varying moment order in *x* and *z* while keeping the 19th order constant in θ.

The results of running these two tests are summarized in Figure 3.

- The evolution of the precision using different orders for the θ coordinate, while keeping constant the orders of moments for the *x* and *z* coordinates both fixed to 19 has been represented using a dashed line. It can be seen how for very low moment orders the performance is very poor, as there is not enough data to estimate 2D moments. However when the order in the θ reaches 7, precision in recognizing the unknown object is above 95%, reaching at the end of the series a maximum value of 98%. This reveals that, in terms of precision, it is sufficient to keep a very low order in the angular moments, given a rich representation in *x* and *z*.
- The evolution of the precision varying the orders for *x* and *z* (taking both the same value in each case), and keeping the order in θ always equal to 19, is represented using a solid line. It can be seen that the precision degrades smoothly but continuously as the moment order decreases. It is then clear that for good results in terms of precision, a rich representation in *x* and *z* is mandatory.

#### 5.1.2. Pose (Angle) Estimation Tests

Following on from the results obtained for the unknown projection images in the tests explained in the previous section, the evaluation is now focused on the accuracy in the estimation of the relative pose of the 3D object (angle θ) that has been used to generate each of the 100 unknown projections. For this, the starting point has been the recognition accomplished in the previous experiment, even if it is wrong. Once the projected 3D object is known, this experiment proceeds by estimating the relative angle between object and observer. Recall that in this simplified case only rotations around the *z* axis are allowed.

Given the structure of vector Γ given in Equation (14), the estimated angle $\theta_{0,u}$ is computed following Equation (22), and then it is compared to the actual angle $\theta_{u}$ that was used to generate each unknown projection. This angle estimation has been carried out, as in the experiment explained above, in two different rounds: first, keeping orders of moments for *x* and *z* fixed to 19 while varying order for θ from 0 to 19, and the second one keeping order for θ in 19 and varying orders for *x* and *z* at the same time from 0 to 19 as well.

The error between the actual angle, $\theta_{u}$, and the estimation, $\theta_{0,u}$ has been computed for each combination of orders in both rounds, and for each one of the one hundred unknown projections, yielding a grand total of 28,000 analysis. Then, for each combination of orders, the RMS errors corresponding to the measured error for each one of the 100 unknowns has been computed:

(64) $$\mathrm{RMS}=\sqrt{\frac{1}{100}\sum_{u=1}^{100}{\left(\theta_{u}-\theta_{0,u}\right)}^{2}}$$

**Figure 4:**
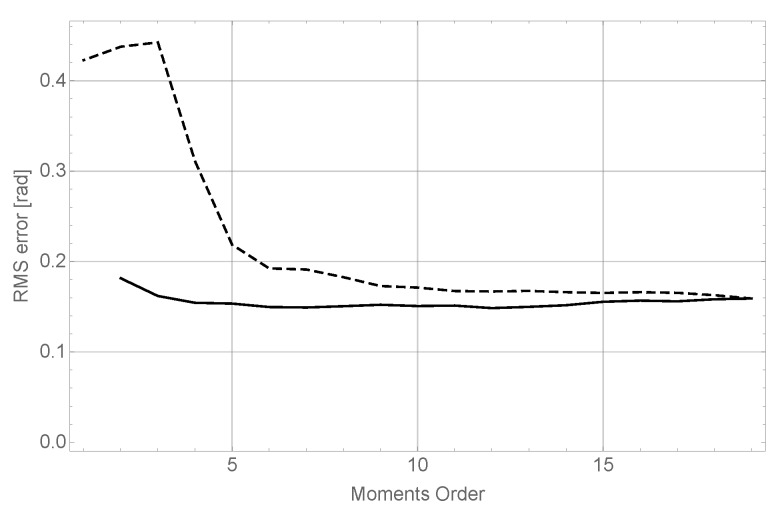
Results of the angle estimation tests. The dashed line shows varying moment order in θ while keeping orders in *x* and *z* constant in 19th, while the solid line shows varying moment order in *x* and *z* while keeping the 19th order constant in θ.

The results of this experiment can be observed in Figure 4.

The conclusions of this experiment confirm the behavior observed in the experiments explained in the previous section. It can be seen (dashed line), that although for very low orders in θ results are very poor, they improve quickly to reach very good values for order 8 without significant enhancements as order 19 is reached. On the other hand, it can be seen as well (dashed line) that the behavior of varying orders in *x* and *z* keeps almost constant; nevertheless, as seen before, maintaining good precision in object recognition requires a high moment order for both coordinates.

In order to make a comparison with algorithms in the literature presenting results working with the same 3D object database, the overall results have been computed separately for each category. Two recently presented algorithms, by Su et al. [30] and Tulsani et al. [31], have been chosen as the basis for comparison; moreover, in those papers the reader can find further references to previous results. Although both of them use median error to evaluate the results and in this paper RMS is used instead, Table 2 provides a good comparison of all these methods.

Quantitatively, the most remarkable result that can be observed is that the RMS value for the complete set has a value of 9.3, being in the same range, or even slightly better, than similar algorithms found in the literature. Nevertheless, it can be observed that the presented algorithm performs worse for some classes.

As an overall conclusion, from these two experiments it can be stated that it is important to keep high orders in *x* and *z*, both in terms of precision and pose estimation, whilst order in θ can be reduced to fairly low values without degrading the outcome of the recognition and pose estimation significantly.

### 5.2. Recognition and Pose Estimation in 4D

In order to test the ability of the algorithm to recognize and estimate the pose of a 3D object, the methodology has been the same as in the former case. The set of test objects has been the same, with the sole difference that, since in this case rotations can be performed over two axes, the 3D grid to define voxelized objects has been increased to a resolution of 60×60×60 (height, width, depth), so it can completely hold the 3D object in any relative position. In these tests, the center of gravity of each 3D object has been placed in the center of the voxel grid, i.e., (30,30,30).

In this case, the projected pseudo-object, defined in 4D, has been built using 10 equally spaced rotations in the range [0,π/2]; once again, this restriction in the total span of the rotations is due to the symmetric nature of the 3D objects in the database. As a result, the dimensions of the 4D object are (60,60,10,10).

The definition of moments along each dimension is found in:

- Cartesian coordinates, i.e., *x* and *z*, and Legendre moments, based on the Legendre polynomials defined in Equation (57), up to the 19th order
- Angular coordinates, i.e., latitude and longitude, and a Fourier basis, as in Equation (58), up to the 9th order. Therefore, the element mnpq of the basis is defined as:
  (65) $$B_{mnpq}=P_{m}\left(x\right)P_{n}\left(z\right)e^{ip\phi }e^{iq\lambda }$$

Computing the volume of data in each stage (3D object, pseudo-object in 4D and moment set), we find that the initial object is defined through 216,000 voxels, that leads to a pseudo-object in 4D of 360,000 elements, represented through 40,000 moments. All this information is gathered in Table 3.

The decision to set the order of moments in each dimension in this manner has been guided by the results shown in the previous section, where it was demonstrated that keeping angular moments in low orders is enough to achieve good results in the pose estimation (in fact, results do not seem to improve when increasing this order). On the other hand, moments in the Cartesian coordinates have been kept at the same order as in the previous section, even though there are more pixels representing each section (recall that now each projection has 60 × 60 pixels, whereas in the previous experiments the size was 60 × 15). This means that having four times the amount of data per projection, the number of moments to represent it is kept the same. The reason is twofold: first, the data volume must kept as low as possible in order to favor computing time and therefore keep the algorithm as close as possible to real time; and second, the resilience of the algorithm should be checked in a situation of data starvation, as the problem now is much more complex than before.

Therefore, in this experimental moment orders have been kept fixed in every case. Once again, it has been split in two stages: object recognition and pose estimation. For the recognition stage, the procedure was the same as in the previous section:

- One hundred different projections of the 3D objects in the database have been generated through randomly selected ϕ and λ angles.
- For each projection, 2D moments up to the 19th order for both x and z have been computed. These moments have been arranged in the vector $S_{obs,u}$.
- For each projection *u* and each object *k* in the database, the estimation of vector $\Gamma_{k,u}$ has been computed, following Equation (39).
- With each computed estimation $\Gamma_{k,u}$, vector $S_{k,u}$ has been reconstructed, using the expression in Equation (34).
- The RMS error between each estimated and observed vectors has been computed, as in Equation (62).
- The recognition is carried out by selecting the object that has generated a reconstruction with minimum RMS error, as in Equation (63).

Finally, for the pose estimation stage, as in previous section, the angles defining the relative pose estimation have been computed following on from the assumption that the previous recognition is right, therefore, the calculations include the false positives. Angles $\phi_{0}$ and $\lambda_{0}$ are computed following Equation (43), and after that, RMS error has been computed for each angle separately, taking into account all the test projections that were generated.

Results for both experiments are summarized in Table 4.

First, it is worth noting the recognition accuracy rate results, which are notably lower than the rate in the cylindrical coordinates problem presented before. The main reason is that many of 3D objects selected, for instance cars or buses, share almost the same look from above, being in every case roughly rectangle-shaped. Therefore, it is hard to precisely recognize which object is seen, leading to some misclassifications.

**Figure 5:**
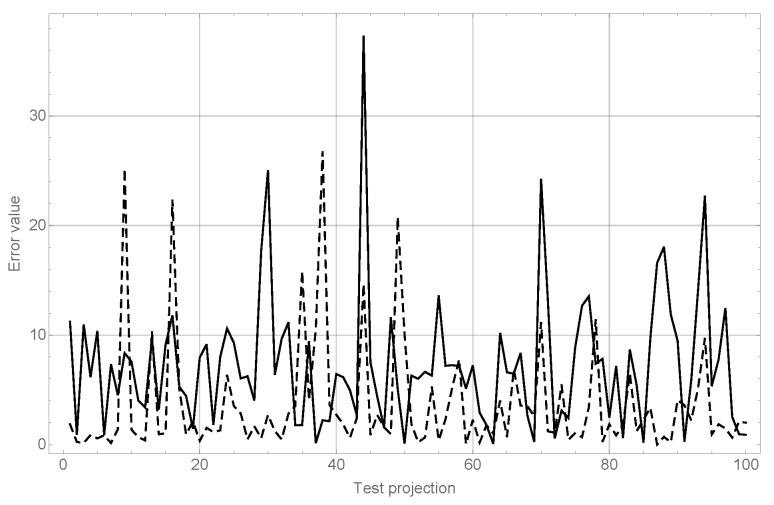
Results of the angle estimation tests for the `Cars’ category: in the solid line, the module of the error series for λ angle, and in the dashed line, the module of the error series for the ϕ angle.

In Table 4 the RMS values are significantly greater than mean values, showing that there could be outliers in the error series that can largely distort results. In Figure 5 the module of the error value for both angle estimations and each test projection in the ‘Cars’ category can be observed in order to illustrate this fact.

As can be seen in Figure 5, wrong recognitions have largely affected the error values, as they tend to generate estimation errors over 20 degrees whilst the trend for objects correctly recognized is to keep errors to under 10 degrees. Table 5 shows the updated results from Table 4 after removing these false positives from the series.

It can be seen that the values obtained for pose estimation are similar to those observed in the reduced cylindrical problem in the previous section.

These results show that the proposed algorithm achieves the same quality as other approaches in the literature in which angle estimation accuracy have been reported, such as in [32] or [17], or a better tradeoff between computing time and quality of estimation, as compared to the work in [14].

### 5.3. Results on Real Images

In order to qualitatively test the algorithm in a real world scenario, in the same way as introduced in [30], images depicting the same car models as those stored in the database were downloaded from the Internet. The only concerns in selecting them were, on one hand, the absence of a strong perspective effect, since the algorithm works under the assumption that projections are orthogonal (parallel), while on the other hand, lens distortions were avoided as well for the same reason. In a real application, these requirements should not be an issue, as the chosen camera can be calibrated previously.

Images were manually segmented and binarized. After that, they were converted to the same size as the projections defining each pseudo-volume stored in the database, i.e., 60×15 pixels, and normalized in the same way as they were, i.e., with the full height and *x* coordinate of the CDG of the object in the middle of the image. After that, 2D moments up to the 19th order in each dimension, *x* and *z*, were computed and passed to the algorithm. The results obtained in this experiment are consistent with the observer evaluation, though, obviously, no ground truth data were available in order to quantitatively evaluate the outcome.

**Figure 6:**
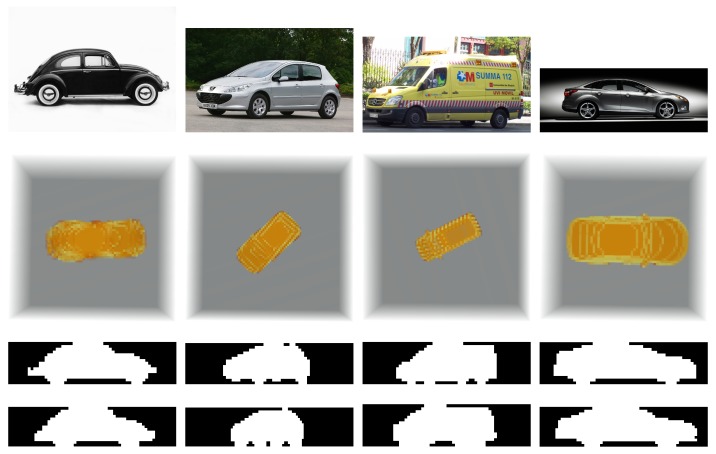
Results of tests on real images. In the first row, the original images as downloaded from the Internet. Second row, 3D objects rotated in the estimated angle and seen from above. Third row, projections after manually segmenting, centering and reducing the scale to 15 × 60 pixels. Fourth row, projections generated applying the estimated rotation angle to the 3D model in the database.

Real images, segmentation results, and projections from the 3D objects in the database using the pose estimation obtained with the algorithm, are collected in Figure 6. Numerical results are gathered in Table 6.

As can be seen, the 3D simulations and the projections generated after them (using the rotation angle obtained with the algorithm) are very close to the segmented images, showing that the estimations, though lacking ground truth data about their true generation, are very consistent with the observed reality.

## 6. Discussion

In this paper, a new method for simultaneous 3D object recognition and pose estimation based on the definition of a set of 4D moments is introduced. The proposed moment set combines Cartesian and angular representations to extract the maximum information of the polynomial approximation achieved by using orthonormal moment bases.

Four-dimensional tensors comprise all the information regarding projection generation, including the 2D Cartesian definition of the projection itself and two angular components to define both latitude and longitude defining the relative pose of the observer.

Cartesian moments allow an efficient representation of object projection, while Fourier moments ease the computation of pose angles through exponential terms. In this way, 3D objects are defined by compact matrices that can be computed off line, leaving recognition and pose estimation stages to rely on simple matrix calculations. Moreover, recognition and pose estimation are accomplished using very low-definition projection images and low-order moments, that allow this algorithm to run in real time.

In addition, this algorithm works for textureless objects, as it is only based on contours, this being a great advantage as opposed to algorithms that need textured objects in order to define keypoints to be matched against known sets.

Results show that both the recognition and pose estimation precision rates are at the level of the state-of-the-art algorithms, proving that this approach is a valuable option for performing both tasks with the advantage of doing it simultaneously.

References yes

## Figures and Tables

**Table 1:** Summary of data representation.

Magnitude	Value
Number of 3D objects	70
Classes of 3D objects	11
Projections per object	20
Δθ between projections	4.5${}^{\circ }$
Moments for *x*	Legendre
Moments for *z*	Legendre
Moments for θ	Fourier
Orders of moments (x,z,θ)	19×19×19
Dimensions in voxels	60×60×15
Dimensions pseudo-volume	60×15×20

**Table 2:** Summary of estimation accuracy for each database category.

	Aero	Bike	Boat	Bottle	Bus	Car	Chair	Table	Mbike	Sofa	Train	TV	Total
**Su et al.**	15.4	14.8	25.6	9.3	3.6	6.0	9.7	10.8	16.7	9.5	6.1	12.6	11.7
**Tuls. TNet**	14.7	18.6	31.2	13.5	6.3	8.8	17.7	17.4	17.6	15.1	8.9	17.8	15.6
**Tuls. ONet**	13.8	17.7	21.3	12.9	5.8	9.1	14.8	15.2	14.7	13.7	8.7	15.4	13.6
**Ours**	7.7	7.6	8.0	-	9.5	10.6	7.5	11.9	12.2	7.6	7.1	11.3	9.3

**Table 3:** Summary of data representation.

Magnitude	Value
Number of 3D objects	55
Classes of 3D objects	11
Projections per object	10 × 10
Δθ between projections	9${}^{\circ }$
Moments for *x*	Legendre
Moments for *z*	Legendre
Moments for ϕ	Fourier
Moments for λ	Fourier
Orders of moments (x,z,ϕ,λ)	19×19×9×9
Dimensions in voxels	60×60×60
Dimensions, pseudo 4D object	60×60×10×10

**Table 4:** Summary of recognition and pose estimation results.

Magnitude	Value
Precision in recognition	91%
RMS in ϕ estimation	11.93${}^{\circ }$
Mean error in ϕ estimation	7.72${}^{\circ }$
RMS in λ estimation	13.59${}^{\circ }$
Mean error in λ estimation	9.21${}^{\circ }$

**Table 5:** Results computed removing false positives.

Magnitude	Value
RMS in ϕ estimation	10.37${}^{\circ }$
Mean error in ϕ estimation	6.81${}^{\circ }$
RMS in λ estimation	11.77${}^{\circ }$
Mean error in λ estimation	8.04${}^{\circ }$

**Table 6:** Results of tests with real images.

Car Model	Classic VW Beetle	Peugeot 207	Ambulance	2011 Ford Focus
**Rotated Angle**	183.29${}^{\circ }$	42.54${}^{\circ }$	26.35${}^{\circ }$	179.83${}^{\circ }$
